# Characteristics Associated with Adults Remembering to Wash Hands in Multiple Situations Before and During the COVID-19 Pandemic — United States, October 2019 and June 2020

**DOI:** 10.15585/mmwr.mm6940a2

**Published:** 2020-10-09

**Authors:** Julia C. Haston, Gabrielle F. Miller, David Berendes, Ashley Andújar, Brittany Marshall, Jennifer Cope, Candis M. Hunter, Brittany M. Robinson, Vincent R. Hill, Amanda G. Garcia-Williams

**Affiliations:** ^1^CDC COVID-19 Response Team; ^2^Epidemic Intelligence Service, CDC; ^3^Division of Foodborne, Waterborne, and Environmental Diseases, National Center for Immunization and Respiratory Diseases, CDC; ^4^Eagle Medical Services, LLC, Atlanta, Georgia.

Washing hands often, especially during times when one is likely to acquire and spread pathogens,* is one important measure to help prevent the spread of SARS-CoV-2, the virus that causes coronavirus disease 2019 (COVID-19), as well as other pathogens spread by respiratory or fecal-oral transmission (*1*,*2*). Studies have reported moderate to high levels of self-reported handwashing among adults worldwide during the COVID-19 pandemic (*3*–*5*)^†^; however, little is known about how handwashing behavior among U.S. adults has changed since the start of the pandemic. For this study, survey data from October 2019 (prepandemic) and June 2020 (during pandemic) were compared to assess changes in adults’ remembering to wash their hands in six situations.^§^ Statistically significant increases in reported handwashing were seen in June 2020 compared with October 2019 in four of the six situations; the odds of remembering to wash hands was 2.3 times higher among respondents after coughing, sneezing, or blowing their nose, 2.0 times higher before eating at a restaurant, and 1.7 times higher before eating at home. Men, young adults aged 18–24 years, and non-Hispanic White (White) adults were less likely to remember to wash hands in multiple situations. Strategies to help persons remember to wash their hands frequently and at important times should be identified and implemented, especially among groups reporting low prevalence of remembering to wash their hands.

Data from ConsumerStyles fall and summer surveys conducted by Porter Novelli Public Services in October 2019 and June 2020 were analyzed for this study.^¶^ These data are collected by Porter Novelli Public Services through Ipsos’ Knowledge Panel, an online market research panel. This panel is designed to be representative of the noninstitutionalized U.S. population, and panel members are recruited randomly by mail through probability, address-based sampling. Respondents receive points for participating in the panel, which can be used to redeem cash and prizes. The samples from each year were weighted to match the U.S. population across eight characteristics: sex, age, annual household income, race/ethnicity, household size, education, U.S. Census division, and residence in a metropolitan area. Sampling weights were applied to all analyses.

The fall 2019 ConsumerStyles survey was completed by 3,624 participants during October 8–22, 2019, (77.5% response rate); the summer 2020 ConsumerStyles survey was completed by 4,053 participants during June 10–25, 2020, (62.7% response rate). The same handwashing question was asked in both surveys: “In which of these situations/settings are you most likely to remember to wash your hands?” with the following response options provided in a randomized order to each participant: 1) after using the bathroom at home; 2) after using the bathroom in public; 3) after coughing, sneezing, or blowing one’s nose; 4) before eating at home; 5) before eating at a restaurant; and 6) before preparing food at home. Participants were asked to select all options for which they would be likely to remember to wash their hands and could choose as many of the six response options as were applicable. In addition to handwashing, collected data included information about demographic characteristics, household size, annual household income, employment status, and perceived health status. Differences in percentages from 2019 to 2020 were considered statistically significant when confidence intervals were not overlapping. Multivariable logistic regression was used to estimate odds ratios (ORs) for the association between remembering to wash hands and year, adjusting for sex, age group, race/ethnicity, health status, U.S. Census division, annual household income, work status, education, metro status, household size, and marital status. All analyses were performed using Stata (Version 15; Stata Corp LP).

The 2019 and 2020 populations were similar in composition across all demographic and socioeconomic characteristics. Respondents frequently reported remembering to wash hands before preparing food at home in 2019 (86.5%) and 2020 (85.7%) (Table 1), after using the bathroom at home (85.9% and 89.6%), and after using the bathroom in public (95.5% and 94.8%) (Table 2). Respondents less commonly reported remembering to wash hands before eating at home in 2019 (62.8%) and 2020 (74.4%), before eating at a restaurant (55.2% and 70.6%), and after coughing, sneezing, or blowing their nose (53.3% and 71.2%).

**TABLE 1:** Percentage of respondents who reported remembering to wash their hands before eating at home, before eating at a restaurant, and before preparing food at home, before and during the COVID-19 pandemic, by selected characteristics — ConsumerStyles fall and summer surveys, United States, October 2019 and June 2020*

Characteristic	Weighted % (95% CI)					
	Before eating at home		Before eating at a restaurant		Before preparing food at home	
	2019	2020	2019	2020	2019	2020
**Overall**	**62.8 (60.9–64.6)**	**74.4 (72.7–76.1)**	**55.2 (53.3–57.1)**	**70.6 (68.9–72.4)**	**86.5 (85.2–87.8)**	**85.7 (84.3–87.1)**
**Sex**						
Women	63.9 (61.2–66.5)	75.3 (72.9–77.6)	56.5 (53.8–59.2)	73.2 (70.8–78.6)	89.9 (88.2–91.6)	89.6 (87.8–91.5)
Men	61.6 (59.0–64.2)	73.5 (71.1–75.9)	53.9 (51.2–56.6)	67.9 (65.4–70.5)	82.9 (80.9–84.9)	81.5 (79.3–83.7)
**Age group (yrs)**						
18–24	62.3 (53.9–70.7)	70.8 (61.8–78.6)	50.8 (42.2–59.5)	65.2 (56.3–74.0)	85.2 (79.1–91.3)	77.0 (69.1–84.9)
25–34	56.3 (51.5–61.2)	66.7 (62.3–71.2)	50.8 (46.0–55.7)	65.6 (61.1–70.1)	84.5 (81.0–88.0)	81.8 (78.1–85.5)
35–44	62.0 (57.6–66.4)	72.0 (68.3–75.7)	55.4 (50.8–60.0)	69.3 (65.5–73.1)	85.3 (82.2–88.4)	85.2 (82.2–88.2)
45–54	65.5 (61.4–69.7)	75.6 (71.9–79.2)	60.4 (56.1–64.7)	75.0 (71.4–78.6)	87.9 (85.1–90.8)	88.4 (85.7–91.1)
55–64	69.1 (65.9–72.3)	81.1 (78.4–83.8)	61.7 (58.3–65.1)	75.1 (72.1–78.2)	89.6 (87.3–91.8)	90.9 (88.8–92.9)
65–74	61.5 (57.6–65.3)	78.8 (75.5–82.0)	53.5 (49.5–57.5)	74.0 (70.6–77.5)	87.6 (84.9–90.4)	87.8 (85.2–90.3)
≥75	62.6 (57.3–68.0)	78.8 (73.7–84.0)	48.6 (43.0–54.2)	67.2 (61.2–72.7)	83.8 (79.6–88.0)	87.8 (83.5–92.0)
**Race/Ethnicity**						
White, NH	58.0 (55.8–60.1)	71.9 (69.9–73.9)	50.6 (48.4–52.8)	68.6 (66.5–70.7)	86.9 (85.5–88.3)	86.0 (84.4–87.5)
Black, NH	76.6 (71.1–82.1)	80.6 (75.5–85.8)	64.9 (58.7–71.2)	75.1 (69.7–80.4)	86.6 (81.9–91.2)	85.6 (80.9–90.4)
Other, NH	69.0 (61.3–76.7)	81.2 (75.1–87.4)	61.7 (53.7–69.8)	79.0 (72.7–85.2)	84.7 (79.0–90.4)	81.5 (74.5–88.4)
Hispanic or Latino	69.0 (63.7–74.4)	75.9 (71.1–80.6)	62.7 (57.1–68.3)	70.9 (65.8–76.0)	85.8 (81.6–90.0)	86.2 (82.1–90.2)
Multiracial, NH	58.7 (47.7–69.8)	84.8 (76.2–93.4)	63.6 (52.9–74.3)	78.0 (69.1–87.0)	85.9 (73.0–93.2)	91.1 (84.6–97.5)
**Health status^†^**						
Excellent	66.6 (60.8–72.3)	76.3 (71.3–81.3)	55.6 (49.3–61.9)	70.7 (65.1–76.3)	86.5 (82.3–90.6)	88.8 (85.3–92.4)
Very good	65.4 (62.5–68.3)	75.0 (72.4–77.7)	58.5 (55.5–61.5)	71.7 (68.9–74.4)	88.2 (86.2–90.2)	86.1 (83.8–88.4)
Good	60.6 (57.5–63.7)	75.2 (72.5–77.9)	53.2 (50.0–56.4)	71.3 (68.4–74.1)	86.1 (83.8–88.4)	84.7 (82.3–87.0)
Fair	56.7 (51.4–61.9)	70.6 (65.3–75.4)	52.7 (47.4–57.9)	67.1 (62.0–72.2)	83.7 (79.8–87.7)	86.3 (82.3–90.2)
Poor	66.4 (56.0–76.7)	69.6 (58.7–80.5)	49.6 (38.6–60.7)	69.6 (58.7–80.4)	82.8 (74.3–91.4)	80.9 (70.8–91.3)
**U.S. Census division**						
New England	49.5 (40.8–58.1)	73.9 (66.7–81.0)	45.3 (36.7–53.5)	73.4 (66.7–80.1)	87.2 (81.8–92.7)	88.7 (84.4–93.0)
Mid-Atlantic	65.6 (60.7–70.4)	73.4 (68.6–78.1)	57.4 (52.3–62.5)	69.8 (65.0–74.6)	87.9 (84.8–91.0)	87.8 (80.5–89.0)
East-North Central	55.0 (50.1–59.8)	75.0 (70.8–79.2)	44.7 (39.8–49.5)	69.4 (65.0–73.8)	83.2 (79.5–87.0)	84.7 (81.2–88.2)
West-North Central	56.3 (49.4–63.3)	62.1 (55.0–69.2)	51.2 (44.3–58.2)	66.6 (59.8–73.4)	83.5 (77.9–89.0)	83.0 (77.0–89.0)
South Atlantic	66.6 (62.7–70.6)	74.8 (71.0–78.5)	59.0 (54.9–63.2)	71.1 (67.2–75.0)	88.3 (85.6–91.0)	82.9 (79.3–86.4)
East-South Central	63.9 (56.1–71.6)	74.5 (66.9–82.1)	58.1 (49.9–66.3)	69.6 (61.3–77.9)	86.6 (80.9–92.2)	86.3 (79.9–92.6)
West-South Central	69.4 (63.9–75.0)	77.1 (72.4–81.7)	59.5 (53.6–65.3)	73.3 (68.3–78.4)	84.5 (80.0–89.0)	87.0 (83.2–90.8)
Mountain	59.6 (52.6–66.5)	71.5 (64.9–78.0)	54.5 (47.3–61.6)	68.6 (62.0–75.2)	87.4 (82.9–91.9)	88.5 (83.9–93.1)
Pacific	64.6 (59.9–69.3)	78.2 (74.2–82.2)	58.6 (53.7–63.4)	71.7 (67.3–76.1)	88.1 (84.9–91.4)	88.7 (85.3–92.0)
**Annual household income (US$)**						
<25,000	63.2 (57.8–68.5)	73.1 (67.7–78.4)	55.5 (50.0–61.0)	64.9 (59.3–70.6)	81.4 (76.9–85.9)	77.6 (72.3–83.0)
25,000–49,999	66.3 (62.0–70.6)	75.8 (71.6–79.9)	60.1 (55.5–64.7)	71.5 (67.1–76.0)	90.2 (87.9–92.6)	84.4 (80.7–88.2)
50,000–74,999	63.1 (58.6–67.5)	76.0 (72.0–80.1)	54.6 (50.0–59.3)	69.8 (65.5–74.0)	86.8 (83.8–89.8)	87.4 (84.2–90.5)
75,000–99,999	63.7 (58.9–68.5)	73.4 (69.0–77.8)	53.3 (48.3–58.4)	72.7 (68.2–77.3)	89.4 (86.3–92.6)	86.9 (83.5–90.2)
100,000–149,999	58.9 (54.7–63.1)	72.7 (69.0–76.4)	52.9 (48.6–57.2)	72.8 (69.3–76.3)	87.1 (84.2–89.9)	89.1 (86.5–91.6)
≥150,000	63.2 (58.0–68.4)	73.1 (68.6–77.7)	55.0 (49.6–60.4)	74.2 (69.7–78.8)	83.0 (78.6–87.3)	86.7 (83.0–90.4)
**Work status** ^§^						
Working	62.3 (59.9–65.6)	73.7 (71.6–75.9)	55.6 (53.2–58.0)	70.8 (68.6–73.0)	86.2 (84.5–87.9)	85.8 (84.1–87.6)
Not working	63.3 (58.3–68.4)	71.1 (66.2–75.9)	55.4 (50.2–60.6)	68.8 (63.7–73.8)	88.1 (84.7–91.5)	82.6 (78.2–87.0)
Retired	63.9 (60.6–67.2)	79.6 (76.8–82.4)	53.7 (50.2–57.1)	71.7 (68.6–74.8)	85.9 (83.5–88.4)	88.1 (85.8–90.4)
**Education**						
Less than high school	64.0 (56.8–71.1)	72.9 (66.2–76.7)	53.7 (46.2–61.2)	65.9 (58.8–73.1)	85.0 (80.1–89.8)	79.9 (73.7–86.1)
High school	65.5 (62.0–69.0)	77.5 (74.4–80.5)	59.2 (55.6–62.8)	72.1 (68.8–75.4)	87.6 (85.2–90.0)	85.9 (83.2–88.6)
Some college	64.9 (61.6–68.3)	74.0 (70.7–77.3)	56.7 (53.1–60.2)	71.4 (68.0–74.8)	87.2 (84.6–89.8)	85.3 (82.4–88.1)
Bachelor’s degree or higher	58.0 (55.1–60.9)	75.6 (70.1–75.1)	51.0 (48.0–53.9)	70.2 (67.7–72.7)	85.5 (83.4–87.6)	87.7 (85.9–89.5)
**Metro status** ^¶^						
Non-metro	64.2 (59.3–69.0)	69.3 (64.4–74.2)	53.2 (48.0–58.3)	65.2 (60.0–70.4)	88.7 (85.5–91.8)	82.7 (78.4–86.9)
Metro	62.6 (60.6–64.5)	75.2 (73.4–77.0)	55.6 (53.5–57.6)	71.5 (69.6–73.3)	86.2 (84.7–87.6)	86.2 (84.7–87.7)
**Household size**						
1	59.9 (56.1–63.8)	75.7 (72.1–79.3)	53.6 (49.7–57.5)	69.0 (65.2–72.9)	84.7 (82.0–87.5)	81.5 (77.9–85.2)
2	61.0 (58.2–63.7)	74.5 (72.0–77.1)	54.7 (51.8–57.5)	70.6 (67.9–73.2)	56.9 (85.0–88.9)	87.0 (84.9–89.0)
3	62.0 (57.4–66.6)	74.7 (70.5–78.9)	51.9 (47.2–56.6)	70.0 (65.6–74.7)	86.2 (82.9–89.4)	84.6 (80.8–88.5)
4	63.1 (57.9–68.2)	72.0 (67.5–76.5)	58.3 (53.0–63.5)	73.6 (69.3–78.0)	88.0 (84.4–91.6)	88.4 (85.3–94.5)
≥5	70.4 (65.1–75.8)	75.2 (70.2–80.2)	58.9 (53.0–64.7)	69.7 (64.3–75.1)	86.0 (82.0–90.1)	85.1 (80.7–89.6)
**Marital status**						
Married/Living with partner	63.3 (61.2–65.4)	75.7 (73.9–77.6)	55.9 (53.7–58.1)	72.3 (70.4–74.2)	87.9 (86.4–89.3)	86.9 (85.4–88.5)
Single	61.9 (58.4–65.3)	72.2 (69.0–75.4)	54.1 (50.6–57.7)	67.9 (64.5–71.3)	84.3 (81.7–86.8)	83.7 (80.9–86.5)

**Abbreviations:** CI = confidence interval; NH = non-Hispanic.

* Surveys were conducted during October 8–22, 2019 (N = 3,624), and June 10–25, 2020 (N = 4,053).

^†^ Health status was self-reported. Participants answered the question, “In general, would you say your health is…?” and were instructed to choose one answer.

^§^ Work status was defined as working (as a paid employee or self-employed); not working (looking for work, on temporary layoff from a job, disabled, or other); and not working, retired.

^¶^ Metro status was defined by U.S. Office of Management and Budget core-based statistical area.

**TABLE 2:** Percentage of respondents who reported remembering to wash their hands after using the bathroom at home, after using the bathroom in public and after coughing, sneezing or blowing their nose, before and during the COVID-19 pandemic, by selected characteristics — ConsumerStyles fall and summer surveys, United States, October 2019 and June 2020*

Characteristic	Weighted % (95% CI)					
	After using the bathroom at home		After using the bathroom in public		After coughing, sneezing, or blowing nose	
	2019	2020	2019	2020	2019	2020
**Overall**	**85.9 (84.6–87.2)**	**89.6 (88.5–90.8)**	**95.5 (94.6–96.3)**	**94.8 (93.8–95.8)**	**53.3 (51.4–55.2)**	**71.2 (69.5–72.9)**
**Sex**						
Women	88.8 (87.1–90.5)	91.4 (89.8–92.9)	96.5 (95.4–97.6)	94.9 (93.5–96.4)	59.7 (57.0–62.4)	76.6 (74.3–78.9)
Men	82.8 (80.7–84.8)	87.8 (86.1–89.6)	94.4 (93.1–95.7)	94.6 (93.3–95.9)	46.4 (43.7–49.1)	65.4 (62.9–68.0)
**Age group (yrs)**						
18–24	84.6 (78.5–90.8)	88.0 (82.0–94.0)	95.7 (92.1–99.3)	90.7 (85.2–96.2)	48.4 (39.7–57.1)	70.5 (62.0–78.9)
25–34	81.8 (78.1–85.5)	88.0 (84.9–91.0)	93.6 (91.1–96.2)	94.7 (92.4–97.1)	50.0 (45.1–54.9)	64.0 (59.5–68.6)
35–44	85.8 (82.8–88.8)	86.7 (83.9–89.6)	97.3 (95.7–98.8)	94.1 (91.8–96.4)	54.9 (50.3–59.4)	70.9 (67.2–74.7)
45–54	86.4 (43.5–89.3)	91.1 (88.7–93.5)	94.9 (92.9–96.8)	95.3 (93.4–97.2)	61.4 (57.1–65.7)	73.8 (70.2–77.4)
55–64	89.5 (87.5–91.6)	91.5 (89.7–93.4)	95.9 (94.3–97.4)	96.5 (95.2–97.9)	55.5 (52.0–59.1)	74.6 (71.6–77.6)
65–74	87.3 (84.8–89.9)	91.9 (89.8–94.0)	96.1 (94.5–97.6)	96.7 (95.2–98.2)	51.7 (47.7–55.7)	75.3 (72.0–78.7)
≥75	86.1 (82.2–89.9)	91.1 (87.7–94.4)	95.1 (92.7–97.4)	93.5 (89.6–97.1)	44.0 (38.4–49.6)	69.2 (63.7–74.7)
**Race/Ethnicity**						
White, NH	84.4 (82.8–85.9)	89.5 (88.1–90.8)	96.4 (95.6–97.1)	96.1 (95.2–97.1)	49.6 (47.4–51.8)	68.9 (66.8–70.9)
Black, NH	88.0 (83.6–92.5)	91.3 (87.9–94.8)	93.2 (89.6–96.9)	91.9 (88.4–95.4)	65.5 (59.4–71.6)	83.2 (78.8–87.5)
Other, NH	90.0 (85.0–95.1)	89.6 (85.2–94.0)	96.4 (93.5–99.3)	95.7 (92.6–98.8)	50.7 (42.4–59.1)	70.3 (63.1–77.4)
Hispanic or Latino	88.8 (85.1–92.5)	89.0 (85.4–92.7)	93.4 (90.5–96.3)	90.8 (87.3–94.4)	60.2 (54.6–65.9)	72.0 (67.0–77.0)
Multiracial, NH	82.9 (73.1–92.8)	90.6 (82.8–98.5)	92.7 (86.4–99.1)	99.4 (98.2–100.0)	49.2 (38.5–60.0)	73.5 (62.7–84.3)
**Health status^†^**						
Excellent	85.2 (81.0–89.3)	90.1 (86.4–93.9)	95.2 (92.7–97.7)	95.1 (92.1–98.1)	55.6 (49.3–61.9)	71.3 (66.0–76.7)
Very Good	87.8 (85.9–89.7)	89.8 (88.0–91.7)	97.2 (96.2–98.2)	96.2 (94.8–97.6)	55.6 (52.6–58.7)	72.1 (69.4–74.8)
Good	85.7 (83.3–88.0)	89.6 (87.7–91.6)	94.7 (93.1–96.3)	94.5 (92.9–96.1)	50.9 (47.7–54.2)	71.4 (68.6–74.3)
Fair	82.7 (78.6–86.7)	90.6 (87.7–93.5)	94.3 (91.5–97.1)	93.7 (90.9–96.5)	51.6 (46.3–56.8)	69.0 (63.3–73.5)
Poor	81.6 (72.5–90.6)	84.3 (76.4–92.3)	89.7 (82.3–97.0)	87.0 (78.6–95.4)	47.2 (36.2–58.2)	69.4 (58.8–80.1)
**U.S. Census division**						
New England	82.5 (76.0–88.9)	92.3 (88.4–96.3)	95.1 (91.8–98.3)	96.3 (93.9–98.7)	55.7 (47.0–64.4)	77.9 (71.2–84.6)
Mid-Atlantic	89.7 (86.5–93.0)	90.6 (87.4–93.7)	96.9 (95.3–98.5)	94.4 (91.7–97.1)	55.5 (50.3–60.8)	72.7 (68.0–77.4)
East-North Central	83.9 (80.4–87.4)	92.2 (89.8–94.7)	94.3 (91.9–96.8)	96.6 (94.7–98.5)	49.0 (44.1–53.9)	72.2 (67.9–76.4)
West-North Central	79.8 (74.1–85.5)	88.0 (83.5–92.5)	96.6 (93.9–99.4)	96.1 (93.5–98.6)	48.4 (41.4–55.4)	71.6 (65.4–77.8)
South Atlantic	86.0 (83.1–88.9)	88.7 (86.1–91.4)	97.1 (95.5–98.7)	94.4 (92.0–96.8)	56.2 (52.1–60.4)	73.2 (69.5–77.0)
East-South Central	87.0 (81.7–92.2)	82.4 (76.0–88.7)	96.1 (93.2–99.0)	91.5 (86.3–96.7)	60.0 (52.1–68.0)	61.9 (53.2–70.6)
West-South Central	85.7 (81.5–89.9)	89.4 (85.7–93.0)	91.2 (87.5–94.8)	94.3 (91.0–97.5)	55.1 (49.2–60.9)	72.0 (66.9–77.1)
Mountain	85.7 (80.6–90.1)	90.2 (85.8–94.5)	95.8 (92.9–98.7)	95.3 (92.0–98.7)	45.2 (38.1–52.4)	69.9 (63.5–76.2)
Pacific	87.5 (84.3–90.8)	89.7 (86.5–92.8)	95.7 (93.6–97.8)	94.2 (91.6–96.8)	52.8 (47.8–57.7)	67.2 (62.8–71.7)
**Annual household income (US$)**						
<25,000	82.1 (77.7–86.5)	85.9 (81.7–90.0)	89.8 (86.3–93.4)	85.7 (81.1–90.3)	57.9 (52.5–63.4)	70.2 (64.6–75.8)
25,000–49,999	89.2 (86.5–91.8)	90.2 (87.4–93.0)	96.8 (95.4–98.2)	95.8 (93.6–98.1)	56.0 (51.3–60.6)	73.5 (69.3–77.7)
50,000–74,999	86.2 (83.2–89.2)	91.0 (88.5–93.5)	95.2 (93.1–97.2)	95.5 (93.4–97.5)	54.2 (49.6–58.9)	71.5 (67.3–75.7)
75,000–99,999	87.1 (83.8–90.4)	90.7 (87.8–93.7)	96.2 (94.5–97.9)	95.8 (93.9–97.7)	53.3 (48.3–58.3)	72.6 (68.1–77.1)
100,000–149,999	85.9 (82.9–88.8)	91.7 (88.4–93.0)	97.7 (96.5–98.9)	97.1 (95.7–98.5)	49.8 (45.6–54.1)	70.9 (67.3–74.5)
≥150,000	87.6 (80.5–88.6)	87.8 (84.3–91.2)	96.7 (94.5–99.0)	95.2 (92.5–97.9)	50.3 (44.9–55.8)	72.6 (68.2–77.1)
**Work status** ^§^						
Working	85.2 (83.5–86.9)	89.3 (87.8–90.8)	95.9 (94.8–96.9)	95.1 (93.9–96.3)	53.2 (50.8–55.6)	70.7 (68.5–72.8)
Not working	86.4 (82.9–89.9)	88.0 (84.5–91.5)	94.8 (92.5–97.1)	93.0 (90.1–95.9)	56.4 (51.2–61.7)	70.7 (65.9–75.6)
Retired	87.7 (85.4–90.0)	92.2 (90.4–94.0)	94.9 (93.4–96.5)	95.4 (93.6–97.1)	50.3 (46.8–53.8)	73.5 (70.4–76.5)
**Education**						
Less than high school	85.9 (81.2–90.7)	88.0 (82.3–92.4)	90.5 (86.5–94.4)	87.8 (82.6–93.1)	58.2 (50.8–65.7)	71.6 (64.7–78.5)
High school	87.7 (85.3–90.1)	90.3 (88.1–92.5)	94.5 (92.8–96.3)	92.9 (90.8–95.0)	59.1 (55.5–62.7)	75.3 (72.1–78.4)
Some college	85.5 (82.8–88.1)	89.0 (86.6–91.3)	96.0 (94.5–97.5)	96.2 (94.7–97.7)	53.0 (49.4–56.6)	72.5 (69.1–78.8)
Bachelor’s degree or higher	84.7 (82.6–86.7)	90.4 (88.8–91.9)	97.5 (96.6–98.4)	97.3 (96.4–98.3)	46.7 (43.8–49.7)	66.7 (64.1–69.3)
**Metro status** ^¶^						
Non-metro	82.4 (78.5–86.2)	86.4 (82.7–90.0)	96.6 (95.0–98.3)	94.5 (91.5–97.4)	52.3 (47.2–57.4)	68.7 (63.6–73.7)
Metro	86.5 (85.4–87.9)	90.2 (88.9–91.4)	95.3 (94.4–96.2)	94.8 (93.8–95.9)	53.4 (51.4–55.5)	71.6 (69.8–73.4)
**Household size**						
1	84.4 (81.6–87.3)	87.0 (84.0–89.9)	94.3 (92.5–96.2)	92.9 (90.4–95.4)	51.1 (47.2–55.0)	69.5 (65.6–73.4)
2	85.1 (83.1–87.1)	89.7 (87.9–91.5)	96.2 (95.1–97.3)	95.5 (93.9–97.0)	51.3 (48.5–54.1)	71.2 (68.6–73.8)
3	86.3 (83.1–89.6)	90.2 (87.2–93.2)	93.4 (90.7–96.1)	94.6 (92.0–97.1)	54.4 (49.2–59.1)	72.6 (68.5–76.8)
4	85.7 (81.9–89.6)	90.6 (88.0–93.3)	95.4 (93.1–97.8)	96.1 (94.1–98.0)	54.8 (49.4–60.1)	71.9 (67.3–76.5)
≥5	88.8 (85.2–92.5)	90.3 (86.7–93.8)	97.6 (96.0–99.1)	94.0 (90.9–97.0)	56.6 (50.2–62.8)	70.5 (65.2–75.8)
**Marital status**						
Married/Living with partner	86.2 (84.7–87.7)	90.1 (88.8–91.4)	96.0 (95.0–96.9)	95.9 (94.9–96.9)	54.0 (51.8–56.2)	71.5 (69.6–73.5)
Single	85.4 (83.0–87.9)	88.9 (86.6–91.1)	94.7 (93.1–96.3)	93.0 (90.9–95.0)	52.1 (48.6–55.7)	70.7 (67.4–73.9)

**Abbreviations:** CI = confidence interval; NH = non-Hispanic.

* Surveys were conducted during October 8–22, 2019 (N = 3,624), and June 10–25, 2020 (N = 4,053).

^†^ Health status was self-reported. Participants answered the question, “In general, would you say your health is…?” and were instructed to choose one answer.

^§^ Work status was defined as working (as a paid employee or self-employed); not working (looking for work, on temporary layoff from a job, disabled, or other); and not working, retired.

^¶^ Metro status was defined by U.S. Office of Management and Budget core-based statistical area.

In 2020, both men and women more frequently reported remembering to wash hands before eating at home and at a restaurant, and after coughing, sneezing, or blowing their nose than they did in 2019. When stratified by age group, a higher percentage of young adults (aged 18–24 years) in 2020 reported remembering to wash hands after having respiratory symptoms compared with 2019, and higher percentages of adults aged ≥25 years reported remembering to wash hands before eating at home and in a restaurant and after having respiratory symptoms in 2020 than did in 2019. In 2020, White participants more frequently reported remembering to wash hands before eating at home, before eating in a restaurant, after using the bathroom at home, and after having respiratory symptoms than they did in 2019. Non-Hispanic Black (Black) and Hispanic or Latino (Hispanic) participants more frequently reported remembering to wash hands after having respiratory symptoms in 2020 than they did in 2019.

Compared with 2019 responses, the odds of reporting remembering to wash hands before eating at home, before eating in a restaurant, after using the bathroom at home, and after coughing, sneezing, or blowing one’s nose were significantly higher in 2020, after controlling for demographic and socioeconomic factors (aOR = 1.72, 2.01, 1.41, and 2.28, respectively) (Table 3). Regardless of year, men were significantly less likely than were women to remember to wash hands before eating at a restaurant, before preparing food, after using the bathroom at home, and after experiencing respiratory symptoms. In addition, young adults (aged 18–24 years) were less likely to remember to wash their hands before eating in a restaurant, before food preparation, and after having respiratory symptoms than were adults aged 45–74 years. Finally, compared with White participants, Black participants were more likely to remember to wash their hands before eating at home, before eating in a restaurant, after using the bathroom at home, and after experiencing respiratory symptoms. Hispanic participants were more likely than were White participants to remember to wash their hands before eating at home, before eating at a restaurant, and after experiencing respiratory symptoms, regardless of year.

**TABLE 3:** Odds of remembering to wash hands before and after six situations, by respondent characteristics — ConsumerStyles fall and summer surveys — United States, October 2019 and June 2020*

Characteristic	aOR (95% CI)					
	Before eating at home	Before eating at a restaurant	Before preparing food at home	After using the bathroom at home	After using the bathroom in public	After coughing, sneezing, or blowing nose
**Overall, year**						
**2019**	**Referent**	**Referent**	**Referent**	**Referent**	**Referent**	**Referent**
**2020**	**1.72 (1.56–1.89)**	**2.01 (1.84–2.20)**	**0.90 (0.78–1.03)**	**1.41 (1.24–1.60)**	**0.79 (0.63–0.98)**	**2.28 (2.08–2.50)**
**Sex**						
Women	Referent	Referent	Referent	Referent	Referent	Referent
Men	0.94 (0.82–1.06)	0.85 (0.75–0.96)	0.53 (0.44–0.63)	0.67 (0.56–0.80)	0.84 (0.62–1.13)	0.58 (0.51–0.66)
**Age group (yrs)**						
18–24	Referent	Referent	Referent	Referent	Referent	Referent
25–34	0.86 (0.61–1.19)	1.04 (0.75–1.43)	1.26 (0.83–1.92)	0.91 (0.58–1.42)	1.12 (0.56–2.26)	1.02 (0.74–1.42)
35–44	1.09 (0.78–1.52)	1.25 (0.90–1.72)	1.46 (0.95–2.25)	0.97 (0.62–1.54)	1.33 (0.64–2.76)	1.38 (0.99–1.93)
45–54	1.29 (0.92–1.81)	1.56 (1.13–2.16)	1.83 (1.19–2.83)	1.29 (0.81–2.04)	1.35 (0.65–2.81)	1.71 (1.23–2.38)
55–64	1.79 (1.29–2.50)	1.72 (1.25–2.38)	2.53 (1.63–3.94)	1.66 (1.05–2.62)	1.94 (0.90–4.21)	1.54 (1.11–2.13)
65–74	1.34 (0.93–1.92)	1.51 (1.06–2.13)	2.01 (1.23–2.37)	1.39 (0.85–2.26)	2.17 (0.95–4.96)	1.44 (1.01–2.05)
≥75	1.43 (0.95–2.14)	1.14 (0.78–1.67)	1.74 (1.02–2.95)	1.31 (0.76–2.25)	1.34 (0.55–3.27)	1.12 (0.76–1.65)
**Race/Ethnicity**						
White, NH	Referent	Referent	Referent	Referent	Referent	Referent
Black, NH	2.00 (1.56–2.55)	1.60 (1.29–1.99)	1.05 (0.77–1.42)	1.39 (1.01–1.92)	0.61 (0.40–0.92)	2.00 (1.59–2.51)
Other, NH	1.64 (1.19–2.26)	1.60 (1.19–2.14)	0.63 (0.43–0.91)	1.26 (0.81–1.95)	0.73 (0.39–1.40)	1.11 (0.82–1.49)
Hispanic or Latino	1.34 (1.09–1.66)	1.32 (1.08–1.62)	0.96 (0.72–1.27)	1.20 (0.88–1.62)	0.59 (0.40–0.88)	1.39 (1.14–1.71)
Multiracial, NH	1.37 (0.92–2.03)	1.50 (1.04–2.18)	1.11 (0.61–2.03)	0.91 (0.50–1.64)	1.10 (0.41–2.94)	1.12 (0.78–1.60)
**Health status** ^†^						
Excellent	Referent	Referent	Referent	Referent	Referent	Referent
Very good	0.90 (0.71–1.15)	1.01 (0.80–1.27)	0.86 (0.62–1.20)	1.07 (0.78–1.46)	1.33 (0.76–2.34)	0.92 (0.73–1.17)
Good	0.72 (0.57–0.92)	0.84 (0.67–1.07)	0.73 (0.52–1.02)	0.93 (0.67–1.29)	0.99 (0.57–1.73)	0.75 (0.59–0.96)
Fair	0.55 (0.41–0.72)	0.73 (0.56–0.97)	0.72 (0.49–1.05)	0.81 (0.55–1.18)	1.08 (0.82–2.01)	0.62 (0.47–0.82)
Poor	0.67 (0.44–1.04)	0.78 (0.51–1.20)	0.69 (0.39–1.22)	0.68 (0.38–1.22)	0.65 (0.29–1.48)	0.63 (0.41–0.96)
**U.S. Census division**						
New England	Referent	Referent	Referent	Referent	Referent	Referent
Mid-Atlantic	1.34 (0.97–1.85)	1.17 (0.85–1.61)	0.87 (0.56–1.39)	1.30 (0.81–2.10)	1.04 (0.53–2.06)	0.82 (0.59–1.14)
East-North Central	1.06 (0.77–1.44)	0.89 (0.66–1.21)	0.68 (0.44–1.05)	1.03 (0.67–1.60)	1.12 (0.59–2.14)	0.65 (0.48–0.90)
West-North Central	0.85 (0.60–1.21)	1.00 (0.71–1.41)	0.59 (0.36–0.98)	0.78 (0.48–1.26)	1.05 (0.49–2.25)	0.69 (0.49–0.99)
South Atlantic	1.31 (0.96–1.78)	1.22 (0.90–1.64)	0.78 (0.50–1.20)	0.94 (0.61–1.44)	1.15 (0.59–2.24)	0.75 (0.55–1.03)
East-South Central	1.25 (0.85–1.83)	1.19 (0.82–1.74)	0.88 (0.52–1.51)	0.77 (0.46–1.29)	0.79 (0.37–1.68)	0.65 (0.44–0.96)
West-South Central	1.50 (1.07–2.10)	1.24 (0.89–1.73)	0.83 (0.53–1.31)	0.93 (0.59–1.49)	0.83 (0.44–1.59)	0.70 (0.50–0.98)
Mountain	1.08 (0.75–1.53)	1.08 (0.76–1.53)	1.00 (0.61–1.65)	1.01 (0.61–1.68)	1.09 (0.52–2.31)	0.61 (0.42–0.87)
Pacific	1.31 (0.95–1.81)	1.16 (0.85–1.60)	1.11 (0.70–1.75)	1.11 (0.70–1.75)	1.11 (0.57–2.15)	0.66 (0.48–0.91)
**Annual household income (US$)**						
<25,000	Referent	Referent	Referent	Referent	Referent	Referent
25,000–49,999	1.09 (0.86–1.38)	1.23 (0.97–1.55)	1.75 (1.28–2.40)	1.63 (1.19–2.24)	3.74 (2.27–6.16)	1.01 (0.79–1.28)
50,000–74,999	1.00 (0.78–1.28)	1.02 (0.80–1.30)	1.62 (1.17–2.23)	1.41 (1.03–1.94)	2.22 (1.41–3.47)	0.93 (0.73–1.20)
75,000–99,999	0.92 (0.70–1.20)	1.02 (0.79–1.31)	1.77 (1.25–2.52)	1.43 (1.01–2.01)	2.53 (1.57–4.09)	0.95 (0.73–1.24)
100,000–149,999	0.84 (0.65–1.09)	1.02 (0.79–1.30)	1.67 (1.19–2.36)	1.36 (0.97–1.90)	3.13 (1.83–5.38)	0.88 (0.68–1.14)
≥150,000	0.91 (0.68–1.21)	1.10 (0.83–1.46)	1.27 (0.85–1.87)	1.08 (0.74–1.59)	1.85 (0.95–3.60)	0.94 (0.71–1.26)
**Work status** ^§^						
Working	Referent	Referent	Referent	Referent	Referent	Referent
Not working	0.97 (0.79–1.18)	0.98 (0.84–1.19)	1.13 (0.85–1.51)	1.07 (0.81–1.41)	1.48 (0.96–2.29)	0.67 (0.79–1.18)
Retired	1.13 (0.93–1.37)	0.99 (0.82–1.19)	0.93 (0.70–1.75)	1.28 (0.97–1.69)	0.87 (0.55–1.38)	1.01 (0.84–1.21)
**Education**						
Less than high school	Referent	Referent	Referent	Referent	Referent	Referent
High school	1.20 (0.91–1.58)	1.27 (0.97–1.65)	1.23 (0.87–1.73)	1.18 (0.82–1.68)	1.45 (0.93–2.25)	1.06 (0.81–1.40)
Some college	1.09 (0.83–1.44)	1.19 (0.91–1.55)	1.19 (0.83–1.69)	1.01 (0.70–1.44)	2.35 (1.41–3.91)	0.88 (0.67–1.16)
Bachelor’s degree or higher	0.91 (0.68–1.21)	1.00 (0.76–1.31)	1.22 (0.85–1.76)	1.03 (0.71–1.50)	2.94 (1.72–5.05)	0.70 (0.53–0.93)
**Metro status** ^¶^						
Non-metro	Referent	Referent	Referent	Referent	Referent	Referent
Metro	0.98 (0.81–1.18)	1.11 (0.93–1.34)	0.91 (0.71–1.17)	1.19 (0.94–1.51)	0.74 (0.48–1.13)	1.06 (0.88–1.27)
**Household size**						
1	Referent	Referent	Referent	Referent	Referent	Referent
2	0.98 (0.81–1.17)	1.07 (0.90–1.28)	1.38 (1.07–1.78)	1.23 (0.95–1.60)	1.38 (0.91–2.09)	1.10 (0.91–1.32)
3	1.14 (0.92–1.42)	1.06 (0.85–1.31)	1.39 (1.03–1.88)	1.60 (1.17–2.18)	1.23 (0.75–2.00)	1.19 (0.95–1.48)
4	1.06 (0.83–1.36)	1.34 (1.05–1.71)	1.69 (1.19–2.41)	1.58 (1.12–2.24)	1.46 (0.82–2.62)	1.21 (0.95–1.55)
≥5	1.39 (1.07–1.82)	1.25 (0.97–1.61)	1.31 (0.92–1.87)	1.82 (1.24–2.67)	1.75 (0.93–3.28)	1.19 (0.92–1.54)
**Marital status**						
Married/Living with partner	Referent	Referent	Referent	Referent	Referent	Referent
Single	0.88 (0.74–1.03)	0.95 (0.81–1.10)	1.04 (0.83–1.30)	1.09 (0.87–1.36)	1.13 (0.78–1.65)	0.93 (0.79–1.10)

**Abbreviations:** aOR = adjusted odds ratio; CI = confidence interval; NH = non-Hispanic.

* Surveys were conducted during October 8–22, 2019 (N = 3,624), and June 10–25, 2020 (N = 4,053).

^†^ Health status was self-reported. Participants answered the question, “In general, would you say your health is…?” and were instructed to choose one answer.

^§^ Work status was defined as working (as a paid employee or self-employed); not working (looking for work, on temporary layoff from a job, disabled, or other); and not working, retired.

^¶^ Metro status was defined by U.S. Office of Management and Budget core-based statistical area.

## Discussion

The findings in this report suggest that the percentage of U.S. adults who reported remembering to wash their hands in certain circumstances has increased during the COVID-19 pandemic compared with prepandemic levels. In June 2020, more U.S. adults reported remembering to wash their hands after coughing, sneezing, or blowing their nose, before eating in a restaurant, before eating at home, and after using the bathroom at home compared with responses in October 2019. The most substantial increases were in the percentages of those remembering to wash their hands after experiencing respiratory symptoms. Despite these increases, however, fewer than 75% of respondents reported remembering to wash their hands after having respiratory symptoms, before eating in a restaurant, and before eating at home. Efforts are needed to communicate the importance of handwashing during these specific situations as well as before food preparation and after using the bathroom.

In both 2019 (prepandemic) and 2020 (during the pandemic), higher percentages of older adults, women, Black persons, and Hispanic persons reported remembering to wash their hands in multiple situations than did young adults, men, and White adults. Because older adults, Black persons, and Hispanic persons have been disproportionately affected by COVID-19 (*6*), engagement in preventive behaviors by these persons is particularly important. The findings of this study are consistent with other studies conducted during the COVID-19 pandemic (*3*,*7*) and past respiratory pandemics (*8*) that have found an association between self-reported handwashing behavior and demographic factors such as sex and age. Although the current study did not explore the reasons for differences in remembering to wash hands among groups, previous work has indicated that older adults perceive personal risks of COVID-19 to be higher than do younger adults, and women have perceived themselves to be at higher risk of infection during respiratory pandemics than have men (*3*,*8*). Also, men and younger adults have less knowledge about symptoms and transmission compared with other groups (*7*), which might affect their handwashing behaviors.

The findings in this report are subject to at least six limitations. First, the cross-sectional design does not allow for assessment of whether the changes in reported remembering to wash hands was directly related to the pandemic or whether respondents might have been influenced by other factors, such as community hygiene promotion activities. However, the same question was asked using the same platform and data collection strategy, which facilitated comparisons over time. Second, the use of overlapping confidence intervals to determine whether the difference between years was statistically significant might result in false negatives, indicating that characteristics did not statistically differ from 2019 to 2020. This methodology is a very conservative approach intended to assess the relationship before estimating aORs. Third, despite weighting to make survey responses nationally representative, persons who agree to participate in online surveys could differ systematically from other members of the public. Fourth, the survey relied on self-report, which could be affected by recall bias or social desirability bias (*9*), resulting in falsely lowered or elevated percentages of those reporting remembering to wash their hands. Fifth, this survey did not assess whether participants had access to handwashing supplies, which might affect the ability to wash one’s hands frequently. Finally, the survey question did not specify how handwashing was performed (e.g., with soap and water) and did not consider hand sanitizer use, which is a recommended method of hand hygiene if soap and water are unavailable.

These findings underscore the importance of promoting frequent handwashing during the ongoing COVID-19 pandemic, especially after coughing, sneezing, and blowing one’s nose. Men, young adults, and White adults continue to be less likely to remember to wash their hands, despite improvements made from 2019 to 2020. Additional work is needed to identify strategies to remind and motivate persons to wash their hands, not only for the prevention of COVID-19, but also to reduce transmission of other infectious diseases transmitted via respiratory or fecal-oral routes. Strategies that have been used in the past to promote handwashing have included active and passive hygiene education, provision of handwashing supplies, environmental cues, and health communication (*2*). These types of efforts should be tailored to resonate with men, young adults, and White adults and continue to specify important times when persons should wash their hands, such as before eating and after coughing, sneezing, or blowing their nose.

**Summary**

**What is already known about this topic?**

Hand hygiene is one important measure to prevent the spread of COVID-19 and other pathogens.

**What is added by this report?**

U.S. adult Internet survey respondents in June 2020 were more likely to remember to wash their hands after experiencing respiratory symptoms, before eating in a restaurant, and before eating at home than were October 2019 survey respondents. Despite improvements, <75% of survey respondents reported remembering to wash their hands in these situations in 2020.

**What are the implications for public health practice?**

Public health efforts should promote frequent handwashing for all, with attention to tailoring messaging to men, young adults, and non-Hispanic White adults. Particular focus should be placed on encouraging handwashing at important times such as before eating and after experiencing respiratory symptoms.

## Footnotes

* https://www.cdc.gov/handwashing/when-how-handwashing.html.

† https://doi.org/10.1101/2020.04.01.20050039.

§ The six situations included the following: after using the bathroom at home; after using the bathroom in public; after coughing, sneezing, or blowing one’s nose; before eating at home; before eating at a restaurant; and before preparing food at home.

¶ https://styles.porternovelli.com/consumer-youthstyles/.
